# Prevalence of Non-responders for Glucose Control Markers after 10 Weeks of High-Intensity Interval Training in Adult Women with Higher and Lower Insulin Resistance

**DOI:** 10.3389/fphys.2017.00479

**Published:** 2017-07-06

**Authors:** Cristian Álvarez, Rodrigo Ramírez-Campillo, Robinson Ramírez-Vélez, Mikel Izquierdo

**Affiliations:** ^1^Department of Physical Activity Sciences, Universidad de Los LagosOsorno, Chile; ^2^Research Nucleus in Health, Physical Activity and Sports, Universidad de Los LagosOsorno, Chile; ^3^Centro de Estudios en Medición de la Actividad Física, Escuela de Medicina y Ciencias de la Salud, Universidad del RosarioBogotá, Colombia; ^4^Department of Health Sciences, Public University of Navarra and Centro de Investigación Biomédica en Red (CIBER) en Fragilidad y Envejecimiento Saludable del Instituto de Salud Carlos IIIPamplona, Spain

**Keywords:** high-intensity interval training, non-responders, insulin resistance, women

## Abstract

**Background:** Exercise training improves performance and biochemical parameters on average, but wide interindividual variability exists, with individuals classified as responders (R) or non-responders (NRs), especially between populations with higher or lower levels of insulin resistance. This study assessed the effects of high-intensity interval training (HIIT) and the prevalence of NRs in adult women with higher and lower levels of insulin resistance.

**Methods:** Forty adult women were assigned to a HIIT program, and after training were analyzed in two groups; a group with higher insulin resistance (H-IR, 40 ± 6 years; BMI: 29.5 ± 3.7 kg/m^2^; *n* = 20) and a group with lower insulin resistance (L-IR, 35 ± 9 years; 27.8 ± 2.8 kg/m^2^; *n* = 20). Anthropometric, cardiovascular, metabolic, and performance variables were measured at baseline and after 10 weeks of training.

**Results:** There were significant training-induced changes [delta percent (Δ%)] in fasting glucose, fasting insulin, and homeostasis model assessment of insulin resistance (HOMA-IR) scores in the H-IR group (−8.8, −26.5, −32.1%, *p* < 0.0001), whereas no significant changes were observed in the L-IR. Both groups showed significant pre-post changes in other anthropometric variables [waist circumference (−5.2, *p* < 0.010, and −3.8%, *p* = 0.046) and tricipital (−13.3, *p* < 0.010, and −13.6%, *p* < 0.0001), supra-iliac (−19.4, *p* < 0.0001, and −13.6%, *p* < 0.0001), and abdominal (−18.2, *p* < 0.0001, and −15.6%, *p* < 0.010) skinfold measurements]. Systolic blood pressure decreased significantly only in the L-IR group (−3.2%, *p* < 0.010). Both groups showed significant increases in 1RM_LE_ (+12.9, *p* < 0.010, and +14.7%, *p* = 0.045). There were significant differences in the prevalence of NRs between the H-IR and L-IR groups for fasting glucose (25 vs. 95%, *p* < 0.0001) and fasting insulin (*p* = 0.025) but not for HOMA-IR (25 vs. 45%, *p* = 0.185).

**Conclusion:** Independent of the “magnitude” of the cardiometabolic disease (i.e., higher vs. lower insulin resistance), no differences were observed in the NRs prevalence with regard to improved HOMA-IR or to anthropometric, cardiovascular, and muscle performance co-variables after 10 weeks of HIIT in sedentary adult women. This research demonstrates the protective effect of HIIT against cardiometabolic disease progression in a sedentary population.

## Introduction

Exercise training is a strategy for the prevention and treatment of several inactivity-related metabolic diseases, such as insulin resistance (Álvarez et al., 2014) and type 2 diabetes mellitus (T2DM) (Alvarez et al., 2016). Similarly, exercise-based interventions, including resistance training (RT), together with pharmacological, and dietary interventions, represent the cornerstones of T2DM management (ADA, 2011). In addition to the beneficial effects on glycemic control (Umpierre et al., 2013) and other risk factors of T2DM (Chudyk and Petrella, 2011; Figueira et al., 2014), physical exercise is effective in improving muscle strength (Dunstan et al., 2002), cardiovascular function (Cano-Montoya et al., 2016), and functional capacity (Cadore and Izquierdo, 2015). In this regard, combining RT and endurance training is an effective intervention to promote overall physical fitness in T2DM patients (Balducci et al., 2012). More recently, high-intensity interval training (HIIT, i.e., repeated short bursts of high intensity activity with rest breaks in between each bout of exercise) is a time-efficient exercise modality that has emerged as an alternative to continuous traditional endurance exercise training to improve cardiometabolic health (Gibala et al., 2012).

However, despite the frequent reports of “average” exercise-related changes, there is wide interindividual variability in the results of exercise training (Astorino and Schubert, 2014). Under the same stimulus, some subjects, termed responders (R), achieve benefits after training, while others, termed non-responders (NRs), show an unchanged or worsened response (Bouchard et al., 2012; Bonafiglia et al., 2016; Álvarez et al., 2017). In the literature, this phenomenon has been characterized using several terms, such as low/high responders (Davidsen et al., 2011), non-responders/responders (Sisson et al., 2009), or as an adverse response (Bouchard et al., 2012); in these studies, similar but slightly different methodological criteria have been applied for identifying R and NRs. Genetic (Stephens et al., 2015) and environmental factors (Bouchard and Rankinen, 2001) have been suggested to be responsible for this variability, although not all of the potential environmental factors (e.g., different health status, magnitude of disease, or different mode of exercise training) have been explored.

Furthermore, the prevalence of these unchanged or worsened responses, known as NRs prevalence (i.e., percentage of subjects who do not improve/show a worsened response with regard to a variable), has been reported predominantly after endurance training (Sisson et al., 2009; Bouchard et al., 2012) and RT (Moker et al., 2014; Churchward-Venne et al., 2015). There have been no studies reporting the NRs prevalence associated with risk factors for T2DM after HIIT, which has been shown to improve anthropometric, cardiovascular, metabolic, and performance variables in different cohorts (Astorino and Schubert, 2014; Alvarez et al., 2016). For example, in one study of insulin resistance adult women, there were reductions of −12 to −14% in fasting glucose, −27 to −37% in fasting insulin and ~40% in homeostasis model assessment of insulin resistance (HOMA-IR) scores after 8 weeks of HIIT (Álvarez et al., 2014). In another study of T2DM subjects, there was a decrease of ~14% in fasting glucose, with additional decreases of ~4 mmHg in blood pressure, ~2% in body mass, ~4% in waist circumference, and ~19% in subcutaneous fat after 16 weeks of HIIT (Alvarez et al., 2016). Notably, another study showed that only 2 weeks of HIIT decreased the average 24 h fasting glucose level by approximately −13% (Little et al., 2011). Finally, a study of subjects with poor glucose control showed an improvement (−12%) in the area under the curve for the oral glucose tolerance test (OGTT) results and a 4.2 kg decrease in fat mass after 10 weeks of HIIT (Mancilla et al., 2014).

Latin America has experienced an epidemiological transition characterized by an increasing burden of cardiometabolic disease due to physical inactivity and shifts in diet and lifestyle patterns (Rivera et al., 2014). Evidence in Chilean adults suggests similar associations between low physical activity levels and cardiometabolic risk factors and between health status and overweight/obesity (Vio et al., 2008). Thus, the aim of the present study was to assess the effects 10 weeks of HIIT and the NRs prevalence (as indicated by glucose control variables) in groups with higher and lower levels of insulin resistance. A second aim was to assess other anthropometric, cardiovascular, and performance variables. We hypothesized that independent of the magnitude of the metabolic disease [i.e., higher (HOMA-IR > 5.0) or lower (HOMA-IR < 3.0) levels of insulin resistance], there would be no differences in the NRs prevalence for changes in glucose control parameters after HIIT between women with higher and lower levels of insulin resistance using the HOMA-IR criteria.

## Materials and methods

### Participants

The first stage of the study was to recruit, using a short telephone survey, adult patients who were previously identified at their last clinical exam as at risk for T2DM and who had dropped out from their regular appointments at the healthcare center. In this first stage, 168 sedentary adult women (aged ≥18 years) with no background of regular exercise training volunteered to be screened.

### Screening and preliminary testing

**Figure 1:**
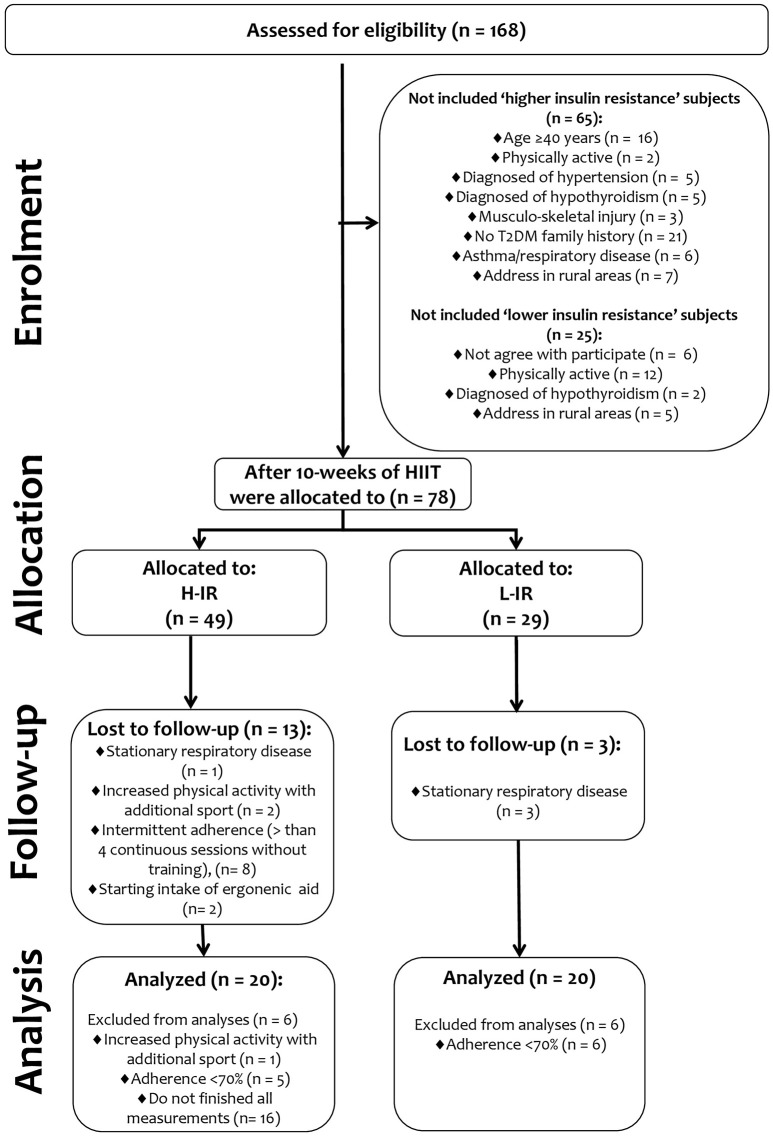
Study design.

First, subjects were screened for insulin resistance based on a HOMA-IR > 2.6 using both fasting glucose and fasting insulin, and after intervention the subjects were separated into two groups and analyzed based on differences in the “magnitude” of insulin resistance (i.e., a group with a higher level of insulin resistance: HOMA-IR > 5.0, and a group with a lower level insulin resistance: HOMA-IR < 3.0). In the first screening before intervention, 65 individuals classified as “higher insulin-resistance” subjects (*n* = 65) were excluded for multiple reasons (16 due to age > 40 years; 2 due to being recently physically active; 5 due to a diagnosis of hypertension; 5 due to a diagnosis of hypothyroidism; 3 due to musculoskeletal injury; 21 due to no history of T2DM; 6 due to stationary asthma/respiratory disease; and 7 due to having a rural address). Similarly, 25 subjects identified as “lower insulin-resistance” subjects (*n* = 25) were also excluded for similar reasons (6 due to age ≥40 years; 12 due to being physically active; 2 due to a diagnosis of hypothyroidism; and 5 due to having a rural address). Finally, 78 screened subjects with a higher level of insulin resistance (*n* = 78) were assigned to 10 weeks of a HIIT program and were analyzed after intervention in two different groups: a group with a higher level of insulin resistance (H-IR, *n* = 20) and a group with a lower level of insulin resistance (L-IR, *n* = 20). None of the subjects were taking oral hypoglycemic medications to improve metabolic control of glucose because they all had been recently diagnosed with insulin resistance by our research team. Subjects with < 70% attendance at training sessions were excluded from all statistical analyses after the intervention; after excluding those subjects, the characteristics of the analyzed subject groups were as follows: H-IR group, age 40 ± 6 years, *n* = 20; L-IR group, age 35 ± 9 years, *n* = 20 (see flow chart in Figure 1). The treatment allocation is described in the flow chart in Figure 1.

All participants were informed about the experimental procedures and about possible risks and benefits associated with participation in the study. Written informed consent was obtained before any of the assessments were performed. The study was conducted in accordance with the Declaration of Helsinki and was approved by the institutional review board for the use of human subjects of the local Ethics Committee of the University of los Lagos (Comité de Revisión Científica y Ética Institucional del Departamento de Ciencias de la Actividad Física de la Universidad de Los Lagos). Characteristics of the study participants are provided in Tables 1, 2.

**Table 1:** Anthropometric characteristic before and after 10-weeks of high-intensity interval training in a higher (H-IR), and lower insulin resistance adult women group (L-IR).

	**H-IR baseline**	**H-IR 10-weeks**	**L-IR baseline**	**L-IR 10-weeks**	***p*-Values*^†^***		***p*-Values^†^**		***P*-values^‡^**	***P*-values^§^**
					**H-IR pre-post**	**Δ% (ES)**	**L-IR pre-post**	**Δ% (ES)**	**H-IR baseline**	**H-IR 10-weeks**
*n* =	20		20							
Age (years)	40 ± 6		35 ± 9							
Height (cm)	155 ± 0.04		158 ± 0.05							
Body mass (kg)	71.4 ± 9.4	69.1 ± 9.1	70.0 ± 7.3	67.8 ± 7.7	0.089	−3.2 (−0.23^#^)	0.061	−3.1 (−0.29^#^)	0.603	0.397
Body mass index (kg/m^2^)	29.5 ± 3.7	28.6 ± 3.5	27.8 ± 2.8	27.0 ± 3.0	0.067	−3.0 (−0.24^#^)	0.189	−2.8 (−0.31^#^)	0.112	0.365
Waist circumference (cm)	98.8 ± 8.2	93.6 ± 8.0	99.7 ± 7.1	95.9 ± 6.7	**<0.010**	−5.2 (−0.61^&^)	**0.046**	−3.8 (−0.52^#^)	0.719	0.167
Tricipital skinfold (mm)	24.7 ± 7.2	21.4 ± 6.9	24.1 ± 6.3	20.8 ± 6.1	**<0.010**	−13.3 (−0.48^#^)	**<0.0001**	−13.6 (−0.57^#^)	0.772	0.399
Supra-iliac skinfold (mm)	31.4 ± 6.4	25.3 ± 7.3	33.7 ± 7.6	29.1 ± 8.1	**<0.0001**	−19.4 (−0.83^&^)	**<0.0001**	−13.6 (−0.55^#^)	0.309	0.112
Abdominal skinfold (mm)	40.5 ± 12.1	33.1 ± 9.4	33.2 ± 7.4	28.0 ± 6.0	**<0.0001**	−18.2 (−0.65^&^)	**<0.010**	−15.6 (−0.85^&^)	**0.028**	0.126

Data are means and ± SD; Delta changes (Δ%) = (10-weeks ^*^ 100/baseline). ES, effect size. Bold values denotes significant differences in all the respectives comparisons.

† *Analyzed by Repeated Measures group × time*.

‡ *Analyzed by ANOVA one-way*.

§ *Analyzed by Bonferroni post-hoc*.

# *Indicates “small” standardized ES at level p ≤ 0.05*.

& *Indicates “moderate” standardized ES at level p ≤ 0.05*.

**Table 2:** Cardiovascular, metabolic, and performance characteristics of the subjects before and after 10-weeks of high-intensity interval training in a group of adult women with a higher level of insulin resistance: HOMA-IR > 5.0 (H-IR), and a group with a lower level insulin resistance: HOMA-IR < 3.0 (L-IR).

	**H-IR baseline**	**H-IR 10-weeks**	**L-IR baseline**	**L-IR 10-weeks**	***p-*****Values**^*†*^		***p-*****Values**^*†*^		***p*-Values^‡^**	***P*-values^§^**
					**H-IR pre-post**	**Δ% (ES)**	**L-IR pre-post**	**Δ% (ES)**	**H-IR vs. L-IR baseline**	**H-IR vs. L-IR post**
*n* =	20		20							
**CARDIOVASCULAR**										
Systolic blood pressure (mmHg)	127 ± 4	124 ± 3	125 ± 4	121 ± 4	0.059	−2.3 (−1.09^&^)	**<0.010**	−3.2 (−0.95^&^)	0.065	0.134
Diastolic blood pressure (mmHg)	74 ± 6	73 ± 6	72 ± 4	71 ± 4	0.076	−1.3 (−0.29^#^)	0.070	−1.3 (−0.16^#^)	0.083	0.377
**METABOLIC**										
Fasting glucose (mg/dL)	113 ± 7	103 ± 6	93 ± 4	91 ± 5	**<0.0001**	−8.8 (−1.65^¶^)	0.179	−2.1 (−0.50^#^)	**<0.0001**	**<0.0001**
Fasting insulin (μU/dL)	20.0 ± 4.7	14.7 ± 6.6	12.4 ± 2.7	10.8 ± 2.8	**<0.0001**	−26.5 (−1.06^&^)	0.145	−12.9 (−0.57^#^)	**<0.0001**	**<0.0001**
HOMA-IR	5.6 ± 1.6	3.8 ± 2.0	2.9 ± 0.7	2.4 ± 0.7	**<0.0001**	−32.1 (−1.23^¶^)	0.165	−17.2 (−0.62^&^)	**<0.0001**	**<0.0001**
**PERFORMANCE**										
1RM_LE_ (kg)	31 ± 3	35 ± 5	34 ± 4	39 ± 4	**<0.010**	+12.9 (0.96^&^)	**0.045**	+14.7 (1.25^¶^)	0.068	0.211
1RM_UR_ (kg)	23 ± 3	25 ± 2	22 ± 2	24 ± 3	0.078	+8.6 (0.62^&^)	0.067	+9.0 (0.44^#^)	0.193	0.267

Data are means and ± SD; Delta changes (Δ%) = [10-weeks ^*^ 100/beseline]; HOMA-IR, homeostasis model assessment of insulin resistance; 1RM_LE_, one-maximum repetition of leg extension; 1RM_UR_, one-maximum repetition of upper row.

*Bold values denotes significant (p < 0.05) differences in all the respectives comparisons*.

† *Analyzed by Repeated Measures group × time*.

‡ *Analyzed by ANOVA one-way/or ANCOVA*.

§ *Analyzed by Bonferroni post-hoc*.

# *Indicates “small” standardized ES at level p ≤ 0.05*.

& *Indicates “moderate” standardized ES at level p ≤ 0.05*.

¶ *Indicates “large” standardized ES at level p ≤ 0.05*.

Eligibility criteria included the following: (a) diagnosed with insulin resistance based on the HOMA-IR metabolic marker method and using a cut-off point of HOMA-IR ≥2.6 in a Chilean population (Garmendia et al., 2009), (b) physical inactivity (≤150 min/week of low-moderate physical activity or <75 min/week of vigorous physical activity; O'Donovan et al., 2010), (c) no familial (parents/siblings) history of T2DM, (d) living only in urban areas, and (e) with care provided under the public Chilean healthcare system (i.e., not a private healthcare system). Exclusion criteria included participants with the following characteristics: (a) potential medical or musculoskeletal problems, (b) osteoarthritis, (c) history of ischemic disease, (d) arrhythmia, (e) asthma, (f) chronic obstructive pulmonary disease, and (g) utilization of drugs that modulate metabolic or respiratory control.

### Classification of responder (R) and non-responders (NRs)

Using previous criteria applied in exercise-based interventions (Bonafiglia et al., 2016), the interindividual variability in the response to exercise training of the subjects was used to categorize them as responders (R) or non-responders (NRs) using the typical error measurement (TE). Thus, the TE was calculated for all independents variables 3 weeks before the pre-test measurements as described previously (Álvarez et al., 2017) using the following equation:

(1) $$\begin{array}{r} TE=SD_{diff}/\surd 2 \end{array}$$

where *SD*_diff_ is the variance (standard deviation) of the difference in scores observed between the two repeats of each test. A non-responders participant for HOMA-IR assessments, as well as for all other included co-variables, was defined as an individual who failed to demonstrate a decrease or increase (in favor of beneficial changes) that was greater than two times the TE from zero. A change greater than two times the TE means that there is a high probability (i.e., 12 to 1 odds) that this response is a true physiological adaptation beyond what might be expected to result from technical and/or biological variability (Hopkins, 2000).

### Procedures

#### Anthropometric and cardiovascular assessments

Anthropometric and blood pressure assessments were carried out during the first week of the allocation stage. Body mass was assessed using a digital scale with an accuracy of 0.1 kg (Omron HBF-INTTM, Omron Healthcare Inc., Lake Forest, IL, USA). Height was assessed with a professional stadiometer (Health o Meter™ Professional, Sunbeam Products Inc., Chicago, IL, USA) with an accuracy of 0.1 cm, and body mass index (BMI) was calculated according to the following formula: kg/m^2^. Waist circumference was assessed with an inextensible measuring tape with and accuracy of 0.1 cm (HoechstmassTM, Sulzbach, Germany). Three skinfold measurements of subcutaneous adipose tissue (i.e., tricipital, supra-iliac, and abdominal skinfolds) were assessed using a Langue™ skinfold caliper (Beta Technology Inc., Santa Cruz, California, USA) according to standard protocols (Marfell-Jones et al., 2006).

Systolic and diastolic blood pressure were assessed using an automatic monitor (Omron HEM 7114™, Omron Healthcare Inc., Lake Forest, IL, USA) in triplicate (2-min interval between measurements) after 15 min of rest and with the subjects in a seated position following standard classification procedures (Chobanian et al., 2003).

#### Plasma metabolic markers

The metabolic measurements were carried out in the second week. Subjects arrived at the laboratory of the Riñihue clinic between 8 and 10 in the morning after a 10 h overnight fast. Blood samples (approximately 3.5 mL) were collected in tubes with specific anticoagulant gels for fasting glucose and fasting insulin measurements at baseline and at the 10 week follow-up. Samples were placed on ice and centrifuged at 4,000 rpm (1,700 × g) for 5 min at 4°C. Plasma samples were immediately transferred to pre-chilled microtubes and stored at −20°C for later analysis.

Plasma glucose was analyzed via enzymatic methods using standard kits (Wiener Lab Inc., Rosario, Argentina) on an automatic analyzer (Metrolab 2300 Plus™, Metrolab Biomed Inc., Buenos Aires, Argentina). Fasting insulin was measured via RIA (DPC, Los Angeles, CA, USA). The HOMA-IR index was calculated using the Matthews equation (Matthews et al., 1985): HOMA-IR = [Fasting glucose (mg/dL) × Fasting insulin (μU/dL)]/405). The same blood sampling and preparation procedure was performed at the end of the 10 week follow-up 48 h after the last exercise session in the morning to avoid possible acute effects of exercise.

#### Familiarization with the exercise training program

In weeks 3 and 4, in three sessions, the subjects in both the H-IR and L-IR groups underwent a familiarization period for the HIIT protocol, as well as for the 1RM_LE_- and 1RM_UR_-tests. In the first and second sessions, the subjects were educated about the cycling machines and the free weights, as well as the exercise machine used for the strength test. In the following four sessions, the subjects underwent HIIT.

#### One-repetition maximum test

In week 5, after a familiarization process with the test and before the intervention, both groups performed one-repetition maximum strength tests to establish 1RM_LE_- and 1RM_UR_-values as previously described (Izquierdo et al., 2004). The 1RM_LE_-test involved an exercise machine (OXFORD™, model EE4002, Santiago, Chile), and in the 1RM_UR_-test, free weights with bars were used. In brief, for the 1RM_LE_-test, the subjects began by lifting a load of weights on the machine with both legs. For the 1RM_UR_-test, the subjects adopted a body flexion angle of 90°, grabbed a bar with weights and plates, and with both arms extended, lifted the bar to approximately knee height. The highest load from three attempts per exercise was reported.

### Experimental protocol

The HIIT program was started in the sixth week and was performed three times per week, for a total of 30 sessions, using exercise bikes (OXFORD™, model BE2601, OXFORD Inc., Santiago, Chile). Each participant performed a range of 8–12 cycling intervals during the intervention period. The time of each cycling work interval was 60 s, with 120 s of passive rest (sitiing on the bicycle without movement) between work intervals. This rest period was progressively decreased (2 min weeks 1–2, 1.45 min weeks 3–5, 1.30 min weeks 6–8, and 1.15 min weeks 9–10), reaching a time of 1.15 min in the tenth week. Cycle revolutions were maintained at a range of 50–70 rpm and a speed between 20 and 40 km/h during each work interval. Subjects were required to cycle at levels between 8 and 10 points on a modified 0–10 Borg scale during the work interval (Ciolac et al., 2015). This subjective intensity corresponds to a range of 70–100% of the maximum heart rate according to the Karvonen formula (Karvonen and Vuorimaa, 1988). All subjects had good exercise tolerance, and none of the participants reported an injury. Exercise compliance was 82.0 ± 3% in the H-IR group and 79.3 ± 4% in the L-IR group.

### Statistical analysis

Data are presented as the mean ± standard deviation (SD). Normality and homoscedasticity assumptions for all data were assessed using the Shapiro-Wilk-test and Levene's-test, respectively. The Wilcoxon-test was used for non-parametric data. One-way ANOVA was performed to test differences between groups at baseline. An ANCOVA was conducted to analyze variables that were significantly different at baseline. A repeated-measures ANOVA (group × time) was used to determine differences in all dependent variables between pre- and post-tests using each group × time. A chi-square test (*X*^2^) was used to determine differences between categorical variables for R and NRs by group (H-IR × L-IR). After the intervention, the typical error (TE) were calculated for the pre-post changes for each dependent variable. The subjects were categorized as a R or NRs according to the previously described criteria of a change greater than two times the TE (Bonafiglia et al., 2016). The Bonferroni *post-hoc* test was applied to establish differences among groups. Additionally, Cohen's-test was used to detect effect sizes (ESs), with threshold values at 0.20, 0.60, 1.2, and 2.0 for small, moderate, large, and very large effects, respectively (Hopkins et al., 2009). ES-values are presented as the mean with 95% confidence limits. Odds ratios (ORs) were used to assess differences in dichotomous NRs variables between groups. All statistical analyses were performed with SPSS statistical software version 18 (SPSS™ Inc., Chicago, Illinois, USA). The alpha level was fixed at *p* ≤ 0.05 for determining statistical significance in all cases.

## Results

### Anthropometric measurements

At baseline, there were significant (*p* ≤ 0.05) differences between groups for abdominal skinfold thickness (Table 1). There were significant (*p* ≤ 0.05) pre-post changes [presented as delta percent (Δ%)] in waist circumference (−5.2, −3.8%) and in tricipital (−13.3, −13.6%), supra-iliac (−19.4, −13.6%), and abdominal skinfold thicknesses (−18.2, −15.6%) in both the H-IR and L-IR groups (Table 1).

### Cardiovascular measurements

At baseline, there were no significant (*p* > 0.05) differences between the groups for diastolic or systolic blood pressure (Table 1). After intervention, the L-IR group showed significant pre-post changes in systolic blood pressure (−2.3%) (Table 2), whereas there were no significant changes in diastolic blood pressure in any group (Table 2).

### Metabolic measurements

At baseline, there were significant (*p* ≤ 0.05) differences between the groups for fasting glucose, fasting insulin, and HOMA-IR scores (Table 2). After intervention, there were no pre-post changes in fasting glucose, fasting insulin, or HOMA-IR scores in L-IR group (Table 2). There were significant (*p* ≤ 0.05) pre-post changes [presented as delta percent (Δ%)] in fasting glucose (−8.8%), fasting insulin (−26.5%), and HOMA-IR scores (−32.1%) in the H-IR group (Table 2). The ES-values were high for fasting glucose (−1.65; 95%CI −2.07, −1.22) and HOMA-IR scores (−1.23; 95%CI −1.60, −0.85) in the H-IR group (Table 2).

### Muscle performance measurements

At baseline, there were no significant (*p* ≤ 0.05) differences between groups for 1RM_LE_ and 1RM_UR_ (Table 2). After intervention, there were significant (*p* ≤ 0.05) pre-post changes in 1RM_LE_ in the H-IR (+12.9) and L-IR (+14.7%) groups (Table 2), whereas 1RM_UR_ remained unchanged in both groups. The ES-value was high for 1RM_LE_ (1.25; 95%CI 1.04, 1.45) in the L-IR group (Table 2).

### Differences in NRs prevalence between the H-IR vs. L-IR groups with respect to glucose control variables

**Figure 2:**
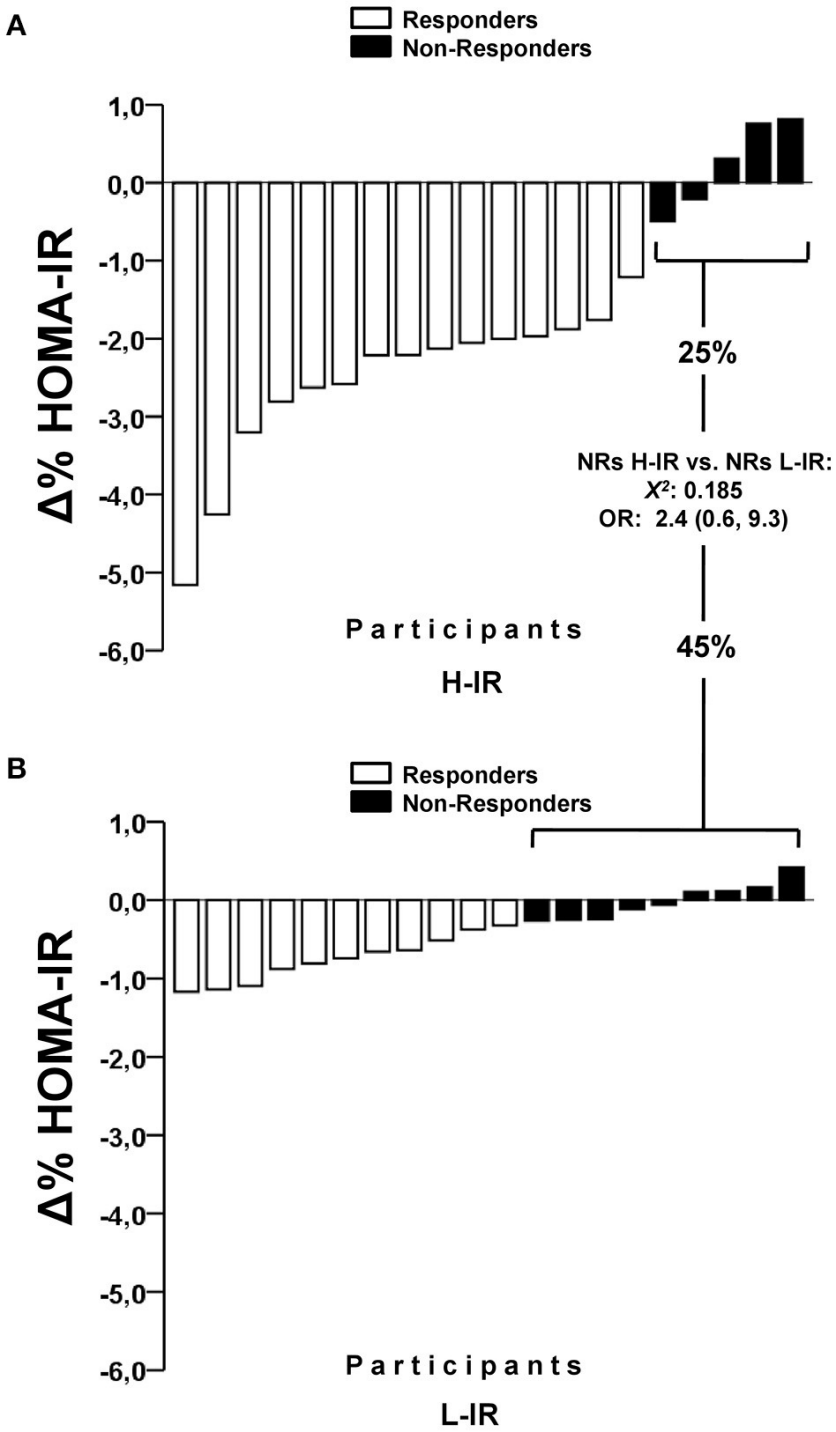
Differences in the prevalence of non-responders to decrease HOMA-IR after high-intensity interval training in a higher insulin resistance (H-IR, *n* = 20), and lower insulin resistance group (L-IR, *n* = 20) of adult women. OR, odds ratios for suffering a non-response.

There were significant differences between the H-IR vs. L-IR groups in NRs prevalence with regard to improved fasting glucose (25.0 vs. 95.0%, *p* < 0.0001) and fasting insulin (25.0 vs. 60.0%, *p* = 0.025). There were no significant differences between the H-IR vs. L-IR groups in NRs prevalence with regard to a decrease in HOMA-IR scores (25.0 vs. 45.0%, *p* = 0.185; Figure 2).

### Differences in the NRs prevalence between the H-IR vs. L-IR groups with respect to other anthropometric, cardiovascular, and performance variables

There were no significant differences between the H-IR vs. L-IR groups in NRs prevalence with regard to improvements in anthropometric parameters (i.e., body mass, BMI, waist circumference, and tricipital, supra-iliac, and abdominal skinfolds), muscle performance (i.e., 1RM_LE_ and 1RM_UR_), or cardiovascular parameters (i.e., systolic/diastolic blood pressure; Table 3).

**Table 3:** Prevalence of non-responders (NRs) on anthropometric, cardiovascular, metabolic, and performance parameters after 10-weeks high-intensity interval training in a group of adult women with a higher level of insulin resistance: HOMA-IR > 5.0 (H-IR), and a group with a lower level insulin of resistance: HOMA-IR < 3.0 (L-IR).

	**Response**	**H-IR**	**L-IR**	**OR**	***p*-Values**
				**(95% CI)**	**NRs H-IR vs. NRs L-IR**
*n* =		20	20		
**ANTHROPOMETRIC**					
Body mass (%/*n*=)	NRs	20.0 (4)	10.0 (2)	0.4 (0.7, 2.7)	0.376
	R	80.0 (16)	90 (18)		
Body mass index (%/*n*=)	NRs	25.0 (5)	10.0 (2)	0.3 (0.5, 1.9)	0.212
	R	75.0 (15)	90.0 (18)		
Waist circumference (%/*n*=)	NRs	5.0 (1)	10.0 (2)	2.1^*^ (0.1, 3.2)	0.548
	R	95.0 (19)	90.0 (18)		
Tricipital skinfold (%/*n*=)	NRs	5.0 (1)	5.0 (1)	1.0 (0.5, 0.9)	0.987
	R	95.0 (19)	95.0 (19)		
Supra-iliac skinfold (%/*n*=)	NRs	30.0 (6)	30.0 (6)	1.0 (0.2, 3.8)	0.944
	R	70.0 (14)	70.0 (14)		
Abdominal skinfold (%/*n*=)	NRs	10.0 (2)	5.0 (1)	0.4 (0.3, 5.6)	0.543
	R	90.0 (18)	95.0 (19)		
**CARDIOVASCULAR**					
Systolic blood pressure (%/n =)	NRs	55.0 (11)	70.0 (14)	1.9 (0.5, 7.0)	0.327
	R	45.0 (9)	30.0 (6)		
Diastolic blood pressure (%/*n*=)	NRs	90.0 (18)	100 (20)	2.1^*^ (1.5, 2.9	0.147
	R	10.0 (2)	0 (0)		
**METABOLIC**					
Fasting glucose (%/*n*=)	NRs	25.0 (5)	95.0 (19)	4.0^*^ (6.2, 14.4)	**<0.0001**
	R	75.0 (15)	5.0 (1)		
Fasting insulin (%/*n*=)	NRs	25.0 (5)	60.0 (12)	4.5 (1.1, 4.3)	**0.025**
	R	75.0 (15)	40.0 (8)		
**PERFORMANCE**					
1RM_LE_ (%/*n*=)	NRs	10.0 (2)	0 (0)	0.4 (0.3, 0.6)	0.147
	R	90.0 (18)	100 (20)		
1RM_UR_ (%/*n*=)	NRs	20.0 (4)	35.0 (7)	2.1^*^ (0.5, 9.0)	0.288
	R	80.0 (16)	65.0 (13)		

*Data are percentage, %/n = number of cases. 1RM_LE_, one-maximum repetition of leg extension; 1RM_UR_, one-maximum repetition of upper row. Bold values denotes significant (p < 0.05) differences between NRs from H-IR vs. NRs from the L-IR group at level (p < 0.05)*.

* *Denotes a high risk (>2-fold) for suffering a non-response*.

The OR analysis for NRs prevalence detected a high risk of being a NRs (>2-fold) associated with improved waist circumference (OR: 2.1, 95%CI 0.1, 3.2), diastolic blood pressure (OR: 2.1, 95%CI 1.5, 2.9), fasting glucose (OR: 4.0, 95%CI 2.2, 14.4), and 1RM_UR_ (OR: 2.1, 95%CI 0.5, 9.0; Table 3).

## Discussion

The present study was designed to assess the effects 10 weeks of HIIT and NRs prevalence (as indicated by glucose control parameters) in adult women with higher and lower levels of insulin resistance to test if the “magnitude” of a metabolic disease play a role in increasing or decreasing the NRs prevalence. The major findings of this study indicate that (i) HIIT promotes significantly more benefits in training-induced changes in fasting glucose, fasting insulin and HOMA-IR scores in adult women with higher insulin resistance; (ii) the NRs prevalence was significantly different between the H-IR vs. L-IR groups with regard to improve fasting glucose and fasting insulin but not for HOMA-IR scores; and (iii) both the H-IR and L-IR groups experienced similar positive training-induced changes and similar NRs prevalence with regard to anthropometric (body mass, BMI), cardiovascular (systolic/diastolic blood pressure), and muscle strength performance (1RM_LE_, 1RM_UR_) measures.

Several environmental factors related to NRs prevalence have been reported after training interventions. For example, a recent report assessed the effects of RT at different frequencies (3 and 2 days/week) to tests the effect of frequency in older NRs subjects for 12 and 24 weeks. Major differences between both training regimens were found for body mass, which decreased by ~4.5% at 12 weeks and 23% at 24 weeks. Interestingly, other results included increases in type I (+34.5 vs. +29.4%) and type II muscle fibers (+22.7 vs. +21.1%), as well as increasing leg strength in extension exercises (+0.9 vs. +1.17%) at 12 and 24 weeks, with relatively similar results obtained independent of the training frequency. These results indicated that the frequency of training was not necessarily related to the NRs prevalence for some variables (Churchward-Venne et al., 2015).

There is limited evidence about interindividual variability in exercise training with regard to the NRs prevalence in subjects with low glucose control, and there are several methodological differences in studies comparing the NRs prevalences observed in previous studies (Boulé et al., 2005; Gremeaux et al., 2012; Yates et al., 2013; Moker et al., 2014; Winett et al., 2014; Higgins et al., 2015). For example, for glucose control variables, several authors have observed that after 3 months of strength training (2 days/week, 2 strength exercises at maximal effort), the NRs prevalence for improvements in an OGTT in pre-diabetic patients was 44%. In the present study, we found a NRs prevalence of 15 and 25% in the H-IR and L-IR groups, respectively, for decreased fasting glucose, with no significant difference between the groups (to see Table 3; Winett et al., 2014). Regarding HOMA-IR, the HERITAGE study (Boulé et al., 2005) showed that after 20 weeks of endurance training [30–50 min/session, 55–75% maximum oxygen uptake (VO_2_max) for 20 weeks], 42% of subjects were NRs for a decrease in HOMA-IR scores. We found similar results regarding a decrease in HOMA-IR scores, with a NRs prevalence of 15 and 20% for the H-IR and L-IR groups, respectively (Figure 2). Therefore, considering our 10 weeks of HIIT-based exercise vs. the 20 weeks of endurance exercise in the abovementioned study (Boulé et al., 2005), increasing the environmental “volume” factor of exercise may not necessarily be related to a decrease in the NRs prevalence for improved glucose control variables such as HOMA-IR scores. In a different study (Yates et al., 2013), NRs prevalence of 3% was reported for decreased fasting glucose after 12 months of exercise-based intervention. Similarly, when T2DM subjects were tested after 9 months of endurance training, RT or concurrent training in another study (Stephens et al., 2015), 21% of subjects were NRs for decreased glycated hemoglobin, as well as other body composition and protein markers.

Understanding the NRs prevalence after exercise modes such as HIIT and including populations with higher and lower risks of T2DM, such as those with higher and lower levels of insulin resistance, can be useful for designing more efficient exercise interventions: in this case, populations with higher levels of insulin resistance, such as the H-IR group, defined based on fasting glucose and HOMA-IR scores, are less likely to be NRs after 10 weeks of HIIT. This altered baseline, which we termed previously as “higher insulin resistance,” may be in some way related to potential factors for predicting responses in future long-term studies. Collectively, and in combination with previous reports (Hecksteden et al., 2013b), these findings indicate that the “magnitude” of changes in response to an acute exercise session can be a potential factor for predicting responses to chronic exercise-based interventions. In this study, the magnitude of changes in plasma variables after volitional exercise was very similar to results from another study where subjects showed decreased fasting insulin after chronic exercise training [walking/running at 60% peak oxygen consumption (VO_2_peak) for 4 weeks].

Another study (Moker et al., 2014) exploring another co-variable, blood pressure, showed that after 5 months of endurance training (65–80% VO_2_peak, walking/jogging), RT (8–12 repetitions per set, 8 exercises, 70–85% of their one-maximum repetition value), or concurrent training, approximately ~60% of subjects were NRs for a decrease in systolic and diastolic blood pressure. In our study, we found a NRs prevalence of 20 and 15% for decreased systolic blood pressure in the H-IR and L-IR groups, respectively, as well as a more pronounced NRs prevalence of 30 and 45% for decreased diastolic blood pressure in the H-IR and L-IR groups, respectively (Table 3). Because none of the intervention groups were diagnosed with hypertension, we hypothesized that genetic together with environmental factors, such as time of intervention, mode of training, and non-hypertensive baseline profiles, may be responsible for these results. However, these results were more positive after 10 weeks of HIIT than the 60% NRs prevalence observed in the aforementioned study following 5 months of intervention. Thus, the volume of training does not appear to play a role in NRs prevalence for decreases in systolic or diastolic blood pressure. Evidence has shown the benefit in terms of decreased systolic blood pressure after HIIT interventions (Ciolac, 2012); however, in this non-hypertensive cohort, we did not observe significant training-induced changes in systolic or diastolic blood pressure (Table 2). In other studies, there was an ~60% NRs prevalence for decreased systolic or diastolic blood pressure after 6 weeks (Higgins et al., 2015) or 6 months (Moker et al., 2014) of HIIT. Interestingly, a study that explored the magnitude of the changes in blood pressure after an acute exercise session reported that this response can be used as a predictive factor for decreases in blood pressure after long-term exercise training (Hecksteden et al., 2013a).

In this study, we found significant training-induced changes in 1RM_LE_-test results in the H-IR (+12.9%) and L-IR (+14.7%) groups (Table 2). Similarly, we found a 10% NRs prevalence for an increase in 1RM_LE_ results in the H-IR group and no NRs (0%) in the L-IR group (Table 3). However, in previous studies, RT (10–15 repetitions, four sets of leg extension, 60–80% of the one-maximum repetition value) resulted in a minimum NRs prevalence of ~1% for an increase in 1RM_LE_ after 12 and 24 weeks of RT (Churchward-Venne et al., 2015). Additionally, despite the fact that our HIIT mode of training is very different methodologically than what was reported in previous studies, the HIIT protocol was able to increase the strength of the lower limbs. These findings are consistent with a previous HIIT-based study (90 s, 6 bouts, 6 weeks), in which HIIT improved several parameters related to power cycling in the lower limbs in adult men (Ziemann et al., 2011).

We observed different ranges of NRs prevalence for other anthropometric (5–30%), blood pressure (55–100%), metabolic (25–95%), and performance (0–35%) variables. These results are consistent with literature reports for blood pressure (59–60%) (Moker et al., 2014), metabolic (7–44%) (Boulé et al., 2005; Yates et al., 2013; Winett et al., 2014; Osler et al., 2015; Stephens et al., 2015), and performance (1%) variables (Churchward-Venne et al., 2015). Finally, our study has some important limitations. Our sample size was limited, but it is similar to the sample sizes used in other exercise training studies (~10–20 subjects). Additionally, we lacked a true no-exercise control group, and we did not control the physical activity patterns and diet of subjects after training, although subjects were reminded each week to maintain their baseline patterns of activity and food consumption. The strengths of this study were that we included both the effects of HIIT and NRs prevalences for changes in anthropometric, cardiovascular, and metabolic risk factors and in performance variables. We also included a statistical estimate of the ES for each variable studied.

## Conclusion

In summary, independent of the “magnitude” of the cardiometabolic disease (i.e., higher vs. lower insulin resistance), no differences were observed in the NRs prevalence with regards to improved HOMA-IR scores, other anthropometric, cardiovascular, and muscle performance variables after 10 weeks of HIIT in sedentary adult women. This research demonstrates the protective effect of HIIT against cardiometabolic disease progression in a sedentary population.

## Author contributions

CA conceived and designed the project. CA and RR-C reviewed the literature studies and conducted data extraction. CA conducted data analyses. CA, RR-C, and MI were responsible for data interpretation. CA, RR-C, and MI drafted the manuscript, and RR-V, and MI revised it critically for intellectual contributions. CA and RR-C coordinate the study development. All authors reviewed and edited the manuscript. All authors read and approved the final manuscript.

### Conflict of interest statement

The authors declare that the research was conducted in the absence of any commercial or financial relationships that could be construed as a potential conflict of interest. The reviewer AS and handling Editor declared their shared affiliation, and the handling Editor states that the process nevertheless met the standards of a fair and objective review.

## Abbreviations

T2DM: type 2 diabetes mellitus
R: responders
NRs: non-responders
HIIT: high-intensity interval training
H-IR: higher insulin resistance group
L-IR: lower insulin resistance group
BMI: body mass index
HOMA-IR: homeostasis model assessment of insulin resistance
1RM: one repetition maximum strength test
1RM_LE_: one repetition maximum test of leg extension
1RM_UR_: one repetition maximum test of upper row
HbA1c: glycated hemoglobin
OGTT: oral glucose tolerance test
VO_2_peak: maximum peak of oxygen uptake
VO_2_max: maximum oxygen uptake.
